# Predicting over-the-counter antibiotic use in rural Pune, India, using machine learning methods

**DOI:** 10.4178/epih.e2024044

**Published:** 2024-04-13

**Authors:** Pravin Arun Sawant, Sakshi Shantanu Hiralkar, Yogita Purushottam Hulsurkar, Mugdha Sharad Phutane, Uma Satish Mahajan, Abhay Machindra Kudale

**Affiliations:** Department of Health Sciences, School of Health Sciences, Savitribai Phule Pune University, Pune, India

**Keywords:** Antibiotic resistance, Antibiotic, Pharmacy, Machine learning, Algorithm, India

## Abstract

**OBJECTIVES:**

Over-the-counter (OTC) antibiotic use can cause antibiotic resistance, threatening global public health gains. To counter OTC use, this study used machine learning (ML) methods to identify predictors of OTC antibiotic use in rural Pune, India.

**METHODS:**

The features of OTC antibiotic use were selected using stepwise logistic, lasso, random forest, XGBoost, and Boruta algorithms. Regression and tree-based models with all confirmed and tentatively important features were built to predict the use of OTC antibiotics. Five-fold cross-validation was used to tune the models’ hyperparameters. The final model was selected based on the highest area under the curve (AUROC) with a 95% confidence interval (CI) and the lowest log-loss.

**RESULTS:**

In rural Pune, the prevalence of OTC antibiotic use was 35.9% (95% CI, 31.6 to 40.5). The perception that buying medicines directly from a medicine shop/pharmacy is useful, using antibiotics for eye-related complaints, more household members consuming antibiotics, and longer duration and higher doses of antibiotic consumption in rural blocks and other social groups were confirmed as important features by the Boruta algorithm. The final model was the XGBoost+Boruta model with 7 predictors (AUROC, 0.934; 95% CI, 0.891 to 0.978; log-loss, 0.279) log-loss.

**CONCLUSIONS:**

XGBoost+Boruta, with 7 predictors, was the most accurate model for predicting OTC antibiotic use in rural Pune. Using OTC antibiotics for eye-related complaints, higher consumption of antibiotics and the perception that buying antibiotics directly from a medicine shop/pharmacy is useful were identified as key factors for planning interventions to improve awareness about proper antibiotic use.

## GRAPHICAL ABSTRACT

[Fig f2-epih-46-e2024044]

## Key Message

Significant predictors of over-the-counter (OTC) antibiotic use by using machine learning methods (ML) were households that found purchasing medications directly from a pharmacy useful were more likely to consume antibiotics for eye-related complaints, engage in longer durations of antibiotic use, take higher doses of antibiotic medications, and have more household members using antibiotics in rural blocks and other social groups. This study represents initial attempts to employ ML methods to identify predictors of OTC antibiotic use for individual households, which can assist in devising intervention strategies to curb the non-prescription use of antibiotics in rural parts of Pune district, Maharashtra.

## INTRODUCTION

The emergence of antimicrobial-resistant (AMR) bacterial species that are beyond the reach of medical treatment is a consequence of the over-the-counter (OTC) consumption of antibiotics in human and veterinary medicine [1-4]. The misuse and overuse of antibiotics, along with self-medication, have accelerated the rise of AMR in bacteria. According to a World Health Organization report, 50% of antibiotic prescriptions worldwide are inappropriate, with India being one of the largest consumers of these drugs [5-7]. The prevalence of OTC antibiotic practices in India can be linked to its highly privatised healthcare infrastructure, informal sectors, and the widespread availability of retail medical stores that sell medicines without valid prescriptions [1]. Previous studies have indicated that the high volume of antibiotic consumption in India [8] is associated with a lack of public knowledge, resource limitations in rural areas, the close proximity of retail pharmacies to the population, cultural practices, inadequate formal healthcare services, and a weak regulatory framework and law enforcement [1,2,4,9]. In an effort to promote antibiotic stewardship, India has enacted the Drugs and Cosmetics Act, 1940, the Drugs and Cosmetics Rules, 1945, Schedule H1 (an amendment to Schedule H, 2014), and has launched a public awareness campaign known as “Medicines with the Red Line” [1,5,9]. Despite these measures, the OTC sale of antibiotics continues to be a widespread practice in the country. Recently, Kerala became the first state in India to initiate Operation Amrith (“Antimicrobial Resistance Intervention for Total Health”). This operation involves conducting surprise inspections at retail medical shops to curb the OTC sale of antibiotics. Additionally, a toll-free number (18004253182) has been established for the public to report complaints against medical shops. Upon receiving a complaint, it is forwarded to the relevant zonal office for investigation, and prompt departmental action is taken if any violations are found [10].

As a step forward in antibiotic stewardship, global studies have utilised artificial intelligence (AI) and machine learning (ML) methods to predict AMR across various bacterial strains [11,12] and to assess the susceptibility of bacterial species to AMR, guiding antibiotic prescriptions with personalised antibiograms. After training with whole-genome sequencing data, several machine-learning algorithms, such as support vector machines (SVM), logistic regression (LR) models, and random forests (RF), have demonstrated high accuracy in predicting AMR [12,13]. The efficacy of deep learning algorithms in identifying new antibiotics, AMR genes, and AMR peptides has also been recently established [14,15]. Studies employing “off-the-shelf” supervised ML algorithms to create predictive models for antibiotic prescribing have yielded promising results, indicating that ML-based solutions can offer essential tools to assist in antimicrobial prescribing and contribute to the fight against AMR [16-18]. Despite these promising results in controlled environments [16-18], the current literature indicates that the application of predictive models to support clinical decisions in antibiotic prescribing and antimicrobial management remains limited and has not yet fully leveraged the significant advancements in data and algorithm development [11,16]. The research has primarily relied on available secondary datasets for conducting AI and ML analyses, with very few studies situated in low-income and middle-income countries, particularly in India.

However, in addition to hospital and laboratory settings, it is essential to implement antibiotic stewardship interventions in community settings. This approach recognises and addresses the behaviours and preferences of both community members and healthcare providers. Against this backdrop, our study sought to identify predictors of OTC antibiotic use in the rural areas of Pune district, India. By employing ML methods on a primary dataset, our study contributes to the identification of these predictors of OTC antibiotic use.

## MATERIALS AND METHODS

### Study design

For primary data collection, a cross-sectional descriptive study was conducted in 2 blocks, Junnar and Mulshi, of Pune district, Maharashtra, to understand antibiotic usage. These blocks were selected based on their proximity to urban settings, with Junnar being distant and tribal, and Mulshi being closer to Pune City and rural.

### Sampling

Pune district is divided into 2 rural sub-divisions. The first, Shirur, is relatively more distant from urban Pune and includes the Junnar, Ambegaon, Khed, and Shirur blocks. The second, Maval, is more accessible and comprises the Maval and Mulshi blocks. These 2 sub-divisions, consisting of 6 blocks, served as the sampling frame for our study. From these, 2 blocks—Junnar and Mulshi —were randomly selected. Within these blocks, a total of 23 villages were chosen: 12 from Junnar and 11 from Mulshi. These villages were selected based on their higher human and livestock populations, using a proportionate sampling approach that accounted for both human and animal population sizes.

### Data collection

Data collection was conducted in 2 phases within the Pune district of Maharashtra State. The first phase included key informant interviews and focus group discussions. Based on the insights gained from the first phase, 3 distinct semi-structured interview schedules were developed for the second phase. This subsequent phase involved gathering both quantitative and qualitative data through semi-structured interviews to understand the perspectives of community members, farmers, and healthcare and veterinary care practitioners on antibiotic use.

### Variables and datasets

The analysis utilised quantitative data from semi-structured interviews. The outcome or dependent variable, OTC antibiotic use, was defined as a binary variable. It was coded as 0 when doctors prescribed antibiotics and household members obtained them from the pharmacy, and as 1 when individuals purchased antibiotics from the pharmacy without a doctor’s advice. This latter category included instances where antibiotics were self-purchased, used from an old prescription, shared by friends, neighbours, or relatives, or suggested and purchased at the pharmacy.

The analysis included a total of 29 predictor/independent variables, which encompassed (1) socio-demographic characteristics of the households, (2) help-seeking behaviour, (3) causes, duration, dosage, and the number of household members who used antibiotics in the past year, and (4) knowledge, awareness, and perceptions about antibiotics. A detailed description of the predictor/independent variables can be found in Supplementary Material 1.

A total of 458 households participated in the survey. Following the exclusion of missing values and non-responses, 443 households remained for inclusion in the analysis. The dataset was randomly split into a training dataset (70% of cases, n= 311) and a testing dataset (30% of cases, n= 132) for the purpose of selecting predictors and developing ML models. We employed 5-fold cross-validation on the training dataset for hyperparameter tuning to minimise prediction error. The performance of the model was assessed using the testing dataset.

### Statistical analysis

All analyses were conducted in R Studio using R version 4.2.3 (R Foundation for Statistical Computing, Vienna, Austria). The exploratory data analysis utilised a complete dataset, with categorical variables described in terms of counts and percentages (%). To examine the association between categorical predictor variables and OTC antibiotic use, we applied the chi-square test of independence. We considered results statistically significant at p-value ≤ 0.05. We calculated the estimated proportions of OTC antibiotic use and their 95% confidence intervals (CIs) using the method proposed by Agresti-Coull, which was implemented with the “prevalence” package [19]. In the Agresti-Coull’s CI formula,


(1) $$Agresti-Coull\;CI=\tilde{\pi }\pm Z\sqrt{\frac{\tilde{\pi }\left(1-\tilde{\pi }\right)}{n+Z^{2}}}\;where\;\tilde{\pi }=\frac{y+\left(\frac{Z^{2}}{2}\right)}{n+Z^{2}}\;Z=Z_{a/2}\;and\;y=Proportion\;of\;OTC\;antibiotic\;use$$

### Selecting predictors

The predictors of OTC antibiotic use were identified by applying LR, the least absolute shrinkage and selection operator (lasso), and Boruta algorithms to the training dataset using the “Caret” package.

LR employs the Akaike information criterion (AIC) for stepwise predictor selection. It eliminates predictors with a p-value greater than 0.10 and compares the AIC of the reduced model at each step to the AIC of the preceding model. The variables that remain in the LR model with the lowest AIC are considered the final predictors. The lasso algorithm, also referred to as L1 penalised/regularised regression, reduces the regression coefficients of unimportant variables to zero [20]. The predictors/variables with non-zero coefficients of the lasso regression model were selected as the final predictors.

The Boruta algorithm, which is based on the RF approach, generates dummy, or shadow, variables corresponding to each of the dataset’s original predictor or independent variables. It then employs a random forest classifier to compare the original predictors with their shadow counterparts using the mean decrease in accuracy and calculates z-scores. An equality test is used to compare the maximum z-score of the shadow predictors against that of the original predictors. If the z-score of an original predictor exceeds the maximum z-score of its shadow, the predictor is retained in the training dataset; otherwise, both the original and its shadow predictor are removed from the dataset. This iterative process continues until all predictors are classified as “confirmed,” “rejected,” or “tentatively important (tntv)” [21]. The predictors identified by the Boruta algorithm as “confirmed important (cnf)” and “tntv” are collectively referred to as “non-rejected predictors (nonrej).”

RF is an ensemble algorithm based on the “bagging” approach, which stands for “bootstrap averaging.” It constructs multiple independent decision tree classifiers (ntree) using a subset of randomly selected variables and two-thirds of bootstrap sample data. The algorithm then validates the predictions with the remaining one-third of the data, known as “out-of-bag” data. RF combines the predictions from all the decision trees, which are trained in parallel, and determines the final predicted class of the outcome variable by the ‘majority vote’ of all the predictions [22]. The extreme gradient boosting tree (XGBtree) algorithm is another ensemble method that enhances prediction accuracy through gradient boosting. Unlike RF, XGBtree builds decision tree classifiers sequentially, learning from the prediction errors of the preceding tree to minimise the error in the subsequent tree. The final prediction is the sum of all individual tree predictions [23,24]. Both the RF and XGBtree algorithms utilise all available variables/predictors, and variable importance (VarImp) is crucial for understanding the significance of these variables/predictors in the model. However, to effectively plan targeted program intervention strategies to reduce the OTC use of antibiotics, it is essential to identify the most important predictors. Therefore, 3 sets of predictors were employed to develop the RF and XGBtree models: (1) all 29 predictors, (2) nonrej selected using the Boruta algorithm, and (3) confirmed important predictors (cnf) also selected using Boruta [25].

### Developing predictive models

Initially, all 29 variables were included in the comprehensive LR model, and the “glmStepAIC” method was employed for the stepwise selection of predictors. The model that yielded the lowest AIC was deemed the final model, and the predictors that remained were chosen as the final predictors. The hyperparameters of lasso (*λ*), RF (*mtry* and *ntree*), and XGBtree (*nrounds, max_depth, colsample_bytree, learning rate eta, gamma, min_child_weight*, and *subsample*) were tuned using cross-validation. The regression coefficients of the selected variables of stepwise logistic and lasso regression, the variable importance from RF and XGBtree, and the mean variable importance with decisions about predictors from the Boruta algorithm are reported. The training dataset was used for selecting predictors, and 5-fold cross-validation was conducted to tune the hyperparameter of the models with selected predictors.

The selected predictors and the best-tuned hyperparameters were used to construct the StepLog and lasso regression models. The RF and XGBtree models were developed using 3 sets of predictors: all 29 predictors for RF and XGBtree; 9 non-rejected predictors for RF+Boruta (nonrej) and XGBtree+Boruta (nonrej); and 7 confirmed important predictors for RF+Boruta (cnf) and XGBtree+Boruta (cnf), each employing the optimally tuned hyperparameters. Model performance was assessed by calculating various metrics: the area under the curve (AUROC) with a 95% CI using the “PROC” package, log-loss, accuracy, sensitivity, specificity, F1-score, and balanced accuracy, using the “ConfusionTableR” package, all based on the test dataset.


(2) $$\log loss=-\frac{1}{n}\sum_{i=1}^{n}\left[y_{i}*\ln\left(P_{i}\right)+\left(1-y_{i}\right)*\ln\left(1-P_{i}\right)\right]\;where\;y_{i}\;is\;actual\;OTC\;antibiotic\;use\;and\;P_{i}\;is\;predicted\;OTC\;antibiotic\;use$$

### Confusion matrix

**Table:** 

Predicted OTC antibiotic use	Actual OTC antibiotic use		Total
	Yes (1)	No (0)	
Yes (1)	a	b	a+b
No (0)	c	d	a+b
Total	a+c	b+d	n


(3) $$\mathrm{Accuracy}=\frac{a+d}{n}$$

(4) $$\mathrm{Sensitivity}=\frac{a}{a+c}$$

(5) $$\mathrm{Specificity}=\frac{d}{b+d}$$

(6) $$F1-\text{score}=2*\frac{a}{\left(a+b)+(a+c\right)}$$

(7) $$\mathrm{Balanced Accuracy}=\frac{\mathrm{Sencitivity}+\mathrm{Specificity}}{2}$$

### Ethics statement

The study was approved by the Institutional Ethics Committee of Savitribai Phule Pune University (Ref. No. SPPU/IEC/2020/84).

## RESULTS

The socio-demographic profile, along with knowledge and practices regarding OTC antibiotic use in households, is presented in Table 1.

Of the 443 households surveyed, 217 (49.0%) were from the tribal Junnar block and 226 (51.0%) from the rural Mulshi block of Pune district, respectively. In the rural areas of Pune district, the use of OTC antibiotics was 35.9% (95% CI, 31.6 to 40.5). The use of OTC antibiotics was significantly higher for complaints related to the ear, nose, and throat (ENT) at 53.3% (95% CI, 36.1 to 69.8), eyes at 53.6% (95% CI, 42.0 to 64.9), and gastrointestinal system (GIS) at 43.7% (95% CI, 32.7 to 55.2). Additionally, in households where more than 1 person used OTC antibiotics, the usage rate was 46.1% (95% CI, 36.1 to 56.4). A significant 39.9% (95% CI, 34.0 to 46.2) of households spent less than 200 Indian rupees (Rs) on purchasing OTC antibiotics. Moreover, 62.5% (95% CI, 47.0 to 75.8) of households perceived that their health condition either did not improve or deteriorated after using antibiotics. Only 23.8% (95% CI, 15.9 to 34.0) of households were aware that not completing the prescribed antibiotic dosage could lead to a deterioration in health.

A strikingly large proportion of households, 97.5% (95% CI, 92.6 to 99.5), believed that the practice of buying antibiotics directly from the pharmacy was useful.

In the tribal block of Junnar, the use of OTC antibiotics was high, with 75.0% (95% CI, 40.1 to 93.7) for ENT complaints, 52.8% (95% CI, 37.0 to 68.0) for GIS issues, and 33.7% (95% CI, 25.3 to 43.2) for respiratory system-related complaints. In the rural block of Mulshi, OTC antibiotics were consumed by more than 1 person per household in 53.5% (95% CI, 38.9 to 67.5) of cases, for more than 10 days in 47.8% (95% CI, 36.2 to 59.5) of cases, and the use was highest at 69.4% (95% CI, 55.4 to 80.6) for eye-related complaints. In Junnar, 41.0% (95% CI, 29.5 to 53.5) of households reported that antibiotic medications were not affordable, and 35.6% (95% CI, 27.5 to 44.6) spent more than Rs 200 on purchasing these medicines. Meanwhile, in Mulshi, only one-fifth of the households reported the unaffordability of OTC antibiotics. In Junnar, 70.0% (95% CI, 47.9 to 85.7) of households perceived that their health condition was not cured or had deteriorated, 57.9% (95% CI, 36.2 to 76.9) reported problems after consuming the medications, and only 18.5% (95% CI, 7.7 to 37.2) reported that purchasing medicines directly from medicine shops or pharmacies was not useful. However, more than 95% of households in both blocks believed that antibiotics are beneficial for human health.

The regression coefficients and the importance of predictors/features are shown in Table 2.

**Figure 1.**
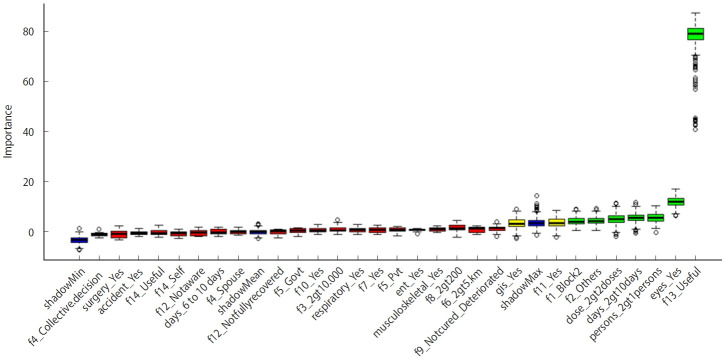
Predictor selection by random forest based Boruta algorithm for predicting over-the-counter antibiotic use in Rural Pune, India.

The perception that buying antibiotics directly from the pharmacy is useful was the most important predictor/feature across all 9 algorithms. Antibiotics used for eye-related complaints ranked as the second most significant predictor. The third most important predictor, according to regression and RF algorithms, was the greater distance of households from healthcare facilities; however, this was not supported by the Boruta algorithm. Rural blocks and membership in other social groups were deemed important by the Boruta algorithm. Additionally, the Boruta algorithm highlighted the significance of having more than 2 persons in a household consuming antibiotics, taking antibiotics for longer than 10 days, and administering more than 2 doses as important factors. Completing the prescribed antibiotic course was also considered a tntv feature by the Boruta argument. The stepwise LR (StepLog) and lasso regression algorithms identified 3 key features: assistance from government healthcare facilities, antibiotics used for respiratory complaints, and the general usefulness of antibiotics for humans as significant predictors. The Boruta algorithm distinguished 7 confirmed and 2 tntv features. The variable importance as determined by the Boruta algorithm is depicted in Figure 1.

The results from evaluating the models’ prediction performance are shown in Table 3.

The final StepLog model had an AIC of 168.52 and included 14 predictors. Its log-loss was 0.378, which was higher than that of other prediction models, and it also had the lowest accuracy (0.864), specificity (0.853), F1-score (0.786), and balanced accuracy (0.872). For the lasso model, the optimally tuned ‘*λ*’ was 0.021, which utilised 9 predictors and achieved a log-loss of 0.326. This model also had the highest sensitivity (0.971) for predicting the use of OTC antibiotics. All RF models were set with ntree= 500. The mtry was 15 for the RF model with all predictors and 2 for the RF+Boruta model, which included 9 non-rejected and 7 confirmed important predictors. The best-tuned hyperparameters for all 3 XGBtree models were: nrounds at 100, max_depth at 20, eta at 0.1, gamma at 0, min_child_weight at 1, and subsample at 1. The hyperparameter colsample_bytree was set at 0.5, 0.7, and 0.8 for the XGBtree, XGBtree+Boruta (nonrej) model with 9 non-rejected predictors, and XGBtree+Boruta (cnf) model with 7 confirmed important predictors, respectively. The RF+Boruta (cnf) and XGBtree+Boruta (cnf) models, both with 7 confirmed important predictors, achieved the highest accuracy (0.909), specificity (0.901), and F1-score (0.864) compared to the other models. The lasso model had the lowest AUROC at 0.902 (95% CI, 0.833 to 0.971). Overall, the StepLog model performed the worst among all the models considered. The XGBtree+Boruta (cnf) model with 7 confirmed important predictors demonstrated the best prediction performance, with the highest AUROC at 0.934 (95% CI, 0.891 to 0.978) and the lowest log-loss at 0.279. Therefore, the XGBtree+Boruta (cnf) model with 7 confirmed important predictors was selected as the final model. The use of OTC antibiotics was predicted for individual households in the rural Pune district by applying this final model.

## DISCUSSION

Our study aimed to identify predictors of OTC antibiotic use in rural communities through the application of ML methods. To the best of our knowledge, this is the first study to employ ML methods to investigate predictors of OTC antibiotic use based on a primary dataset. To minimise geographical and demographic biases, we included multiple study sites, with one located near a city and another situated farther away.

Our study findings indicate that the most significant predictor of OTC antibiotic use was the belief that it is useful to purchase antibiotics directly from pharmacies. This behaviour underscores the cultural and socio-demographic closeness of pharmacists to the rural communities they serve, in contrast to medical doctors. The results also emphasise the need for regulatory interventions to curb OTC antibiotic use, as outlined in Kerala State’s AMR intervention program, Operation Amrith [10]. Additionally, the use of antibiotics for eye-related and GIS complaints emerged as the second most significant predictor, likely due to the higher prevalence of these conditions. In our analysis, the XGBtree+Boruta (cnf) model with 7 predictors was identified as the most accurate in terms of prediction performance. This model outperformed other approaches, including regression models (StepLog, lasso), RF (RF), XGBTree, RF+Boruta (nonrej) with 9 non-rejected predictors, and RF+Boruta (cnf) with 7 confirmed important predictors, as well as XGBtree+Boruta (nonrej) with 9 non-rejected predictors.

This study demonstrated the potential use of ML models for predicting OTC antibiotic use. ML models have proven to be helpful in the medical and health sciences, particularly in the areas of diagnosis and outcome prediction [26]. Previous research has suggested that the application of ML models in the healthcare industry, although still in the early stages, is primarily focused on the early diagnosis of chronic diseases, predicting future disease incidence, conducting epidemiological studies, and facilitating evidence-based decision-making [26-31]. There is also evidence supporting the use of AI and ML models to predict AMR among bacterial species based on whole genome sequencing [12,13,32-35]. As part of antibiotic stewardship efforts, AI and ML have been employed to guide targeted empiric antibiotic prescribing [14,16,18,36], profile and analyse drug resistance, and design targeted drug therapies [37,38] in pharmacometrics [39], and antibiotic discovery [40]. Previously conducted studies in the health and medicine domains have employed several methods, including recursive decision tree-based models, XGBoost [41,42], a fuzzy logic model [43], ADABoost, RF, convolutional neural networks, SVM, LR, lasso regression, and classification and regression trees [44,45].

As this study represents one of the initial attempts of its kind, we contend that employing AI and ML models can assist in the planning and enhancement of public health interventions in other states. This approach could mirror the successes of Operation Amrith in Kerala State [10], potentially increasing the novelty and impact of our study. Additionally, our findings highlight the imperative for more research into the patterns of OTC antibiotic usage that contribute to AMR. Such research should leverage AI and ML to inform targeted antibiotic therapies. Building on the results of our study, we advocate for further investigations that could guide the development of structured health interventions in rural Pune. There is also a pressing need for community-level health education interventions that focus on antibiotic stewardship and the broader implications of AMR.

To summarize, households that found the practice of purchasing medications directly from a pharmacy to be useful were more likely to consume antibiotics for eye-related complaints, engage in longer durations of antibiotic use, take higher doses of antibiotic medications, and have more household members using antibiotics in rural blocks and other social groups. These factors were confirmed as significant predictors of OTC antibiotic use. The XGBtree ML algorithm in conjunction with the Boruta feature selection method, which identified 7 significant predictors, emerged as the best model with the lowest prediction error. Predictions of OTC antibiotic use for individual households can be instrumental in devising intervention strategies aimed at curbing the non-prescription use of antibiotics in the rural areas of Pune district, Maharashtra.

## Figures and Tables

**Figure f2-epih-46-e2024044:**
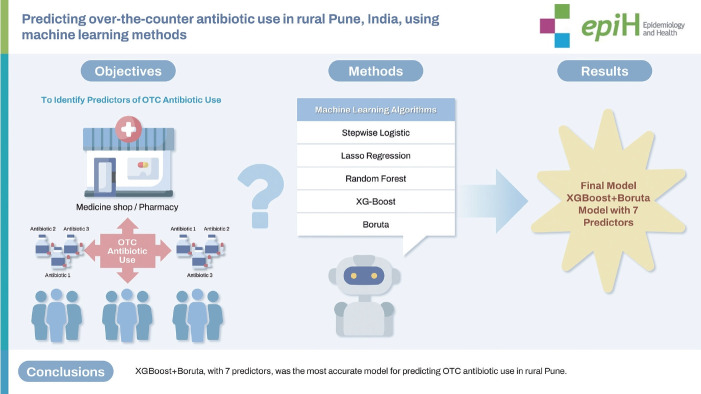


**Table 1.** Socio-demographic characteristics, reasons for antibiotic consumption, and knowledge and awareness about OTC antibiotic use

Predictors	OTC antibiotics use									
	Total participants				Junnar (distant/tribal)			Mulshi (nearer/rural)		
	N	n	% (95% CI)	p-value	N	n	% (95% CI)	N	n	% (95% CI)
Total	443	159	35.9 (31.6, 40.5)							
Block				0.568						
Junnar (distant/tribal)	217	75	34.6 (28.5, 41.1)		217	75	34.6 (28.5, 41.1)	-	-	-
Mulshi (nearer/rural)	226	84	37.2 (31.1, 43.6)		-	-	-	226	84	37.2 (31.1, 43.6)
Community group				0.624						
General	216	80	37.0 (30.9, 43.6)		17	6	35.3 (17.2, 58.8)	199	74	37.2 (30.8, 44.1)
Others	227	79	34.8 (28.9, 41.2)		200	69	34.5 (28.2, 41.3)	27	10	37.0 (21.5, 55.8)
Monthly family income (Indian rupee)				0.413						
<10,000	352	123	34.9 (30.1, 40.1)		183	61	33.3 (26.9, 40.4)	169	62	36.7 (29.8, 44.2)
≥10,000	91	36	39.6 (30.1, 49.8)		34	14	41.2 (26.3, 57.8)	57	22	38.6 (27.0, 51.6)
Responsibility for healthcare decisions				0.188						
Self	150	47	31.3 (24.4, 39.2)		85	25	29.4 (20.7, 39.9)	65	22	33.8 (23.5, 46.0)
Spouse	128	43	33.6 (26.0, 42.2)		60	20	33.3 (22.7, 46.0)	68	23	33.8 (23.7, 45.7)
Close family members	113	45	39.8 (31.3, 49.0)		53	20	37.7 (25.9, 51.2)	60	25	41.7 (30.0, 54.3)
Collective decision	52	24	46.2 (33.3, 59.5)		19	10	52.6 (31.7, 72.7)	33	14	42.4 (27.2, 59.2)
Help from healthcare facilities				0.240						
Government	52	14	26.9 (16.7, 40.3)		29	6	20.7 (9.5, 38.7)	23	8	34.8 (18.7, 55.2)
Private	293	105	35.8 (30.6, 41.5)		115	38	33.0 (25.1, 42.1)	178	67	37.6 (30.8, 44.9)
Both government and private	98	40	40.8 (31.6, 50.7)		73	31	42.5 (31.8, 53.9)	25	9	36.0 (20.2, 55.6)
Distance of healthcare facility (km)				0.295						
<5	145	57	39.3 (31.7, 47.4)		54	18	33.3 (22.2, 46.7)	91	39	42.9 (33.2, 53.1)
≥5	298	102	34.2 (29.1, 39.8)		163	57	35.0 (28.1, 42.6)	135	45	33.3 (25.9, 41.7)
Antibiotics used for complaints										
Ear-nose-throat	30	16	53.3 (36.1, 69.8)	0.039	8	6	75.0 (40.1, 93.7)	22	10	45.5 (26.9, 65.3)
Eyes	69	37	53.6 (42.0, 64.9)	0.001	20	3	15.0 (4.4, 36.9)	49	34	69.4 (55.4, 80.6)
Gastro-intestinal system	71	31	43.7 (32.7, 55.2)	0.136	36	19	52.8 (37.0, 68.0)	35	12	34.3 (20.8, 50.9)
Injury or accident	19	8	42.1 (23.1, 63.8)	0.564	11	6	54.5 (28.0, 78.7)	8	2	25.0 (6.3, 59.9)
Musculoskeletal disorders	24	6	25.0 (11.7, 45.2)	0.253	14	4	28.6 (11.3, 55.0)	10	2	20.0 (4.6, 52.1)
Respiratory system	181	56	30.9 (24.6, 38.0)	0.071	104	35	33.7 (25.3, 43.2)	77	21	27.3 (18.5, 38.2)
Surgery	50	14	28.0 (17.4, 41.8)	0.217	20	4	20.0 (7.5, 42.2)	30	10	33.3 (19.1, 51.3)
Total no. of persons who consumed antibiotics (person)				0.025						
1	354	118	33.3 (28.6, 38.4)		171	57	33.3 (26.7, 40.7)	183	61	33.3 (26.9, 40.4)
>1	89	41	46.1 (36.1, 56.4)		46	18	39.1 (26.4, 53.6)	43	23	53.5 (38.9, 67.5)
Total no. of days antibiotics consumed (day)				0.527						
≤5	239	84	35.1 (29.4, 41.4)		117	47	40.2 (31.7, 49.2)	122	37	30.3 (22.8, 39.0)
6-10	88	36	40.9 (31.2, 51.4)		51	21	41.2 (28.7, 54.8)	37	15	40.5 (26.3, 56.5)
>10	116	39	33.6 (25.7, 42.6)		49	7	14.3 (6.8, 27.0)	67	32	47.8 (36.2, 59.5)
Total no. of tablets/syrups of antibiotics consumed (dose/day)				0.375						
1-2	315	109	34.6 (29.6, 40.0)		147	52	35.4 (28.1, 43.4)	168	57	33.9 (27.2, 41.4)
>2	128	50	39.1 (31.0, 47.7)		70	23	32.9 (23.0, 44.5)	58	27	46.6 (34.3, 59.2)
Antibiotic medicines are affordable				0.258						
No	120	38	31.7 (24.0, 40.5)		61	25	41.0 (29.5, 53.5)	59	13	22.0 (13.2, 34.3)
Yes	323	121	37.5 (32.4, 42.9)		156	50	32.1 (25.2, 39.7)	167	71	42.5 (35.3, 50.1)
Overall money spent on purchasing antibiotic medicines (Indian rupee)				0.052						
<200/-	243	97	39.9 (34.0, 46.2)		99	33	33.3 (24.8, 43.1)	144	64	44.4 (36.6, 52.6)
≥200/-	200	62	31.0 (25.0, 37.7)		118	42	35.6 (27.5, 44.6)	82	20	24.4 (16.3, 34.8)
Perceived effect of antibiotic medicines on health outcomes				<0.001						
Cured	403	134	33.3 (28.8, 38.0)		197	61	31.0 (24.9, 37.7)	206	73	35.4 (29.2, 42.2)
Not cured/deteriorated	40	25	62.5 (47.0, 75.8)		20	14	70.0 (47.9, 85.7)	20	11	55.0 (34.2, 74.2)
Problems after consuming medicines				0.208						
No	403	141	35.0 (30.5, 39.8)		198	64	32.3 (26.2, 39.1)	205	77	37.6 (31.2, 44.4)
Yes	40	18	45.0 (30.7, 60.2)		19	11	57.9 (36.2, 76.9)	21	7	33.3 (17.0, 54.8)
Completed dose of medicine prescribed by doctor				0.213						
No	156	62	39.7 (32.4, 47.6)		57	24	42.1 (30.2, 55.0)	99	38	38.4 (29.4, 48.2)
Yes	287	97	33.8 (28.6, 39.5)		160	51	31.9 (25.1, 39.5)	127	46	36.2 (28.4, 44.9)
Effects/consequences of not completing dose of medicine				0.034						
Incomplete recovery	132	53	40.2 (32.2, 48.7)		72	28	38.9 (28.4, 50.4)	60	25	41.7 (30.0, 54.3)
Health deterioration, partially effective, antibiotic resistance	84	20	23.8 (15.9, 34.0)		50	12	24.0 (14.2, 37.5)	34	8	23.5 (12.2, 40.2)
Not aware	227	86	37.9 (31.8, 44.3)		95	35	36.8 (27.8, 46.9)	132	51	38.6 (30.8, 47.2)
Perception of buying medicines directly from medicine shop/pharmacy				<0.001						
Not useful	322	41	12.7 (9.5, 16.8)		27	5	18.5 (7.7, 37.2)	46	17	37.0 (24.5, 51.4)
Useful	121	118	97.5 (92.6, 99.5)		190	70	36.8 (30.3, 43.9)	180	67	37.2 (30.5, 44.5)
Antibiotics are beneficial for human beings				0.262						
Not beneficial	73	22	30.1 (20.8, 41.5)		153	14	9.2 (5.4, 14.9)	169	27	16.0 (11.2, 22.3)
Beneficial	370	137	37.0 (32.3, 42.1)		64	61	95.3 (86.6, 98.9)	57	57	100.0 (94.6, 100.0)

Responses of “yes” for OTC antibiotic use are shown in the table; Percentages are calculated as n*100/N. OTC, over-the-counter; CI, confidence interval.

**Table 2.** Predictor/feature importance by various machine learning methods for predicting OTC antibiotic use in rural Pune, India

Features	Full logistic	Step wise logistic	Lasso	Boruta		Random forest			XGBtree		
	Regression coefficients			Mean Imp	Decision	RF (all variables)	RF+Boruta (9 nonrej variables)	RF+Boruta (7 cnf variables)	XGBtree (all variables)	XGBtree+Boruta (9 nonrej variables)	XGBtree+Boruta (7 cnf variables)
(Intercept)	-1.30	-0.55	-1.55								
Mulshi (nearer/rural)	0.67	-	-	4.17	Cnf	8.22	2.07	4.69	3.49	1.99	1.23
Social group-Others	0.75	-	-	4.33	Cnf	6.10	4.88	1.69	3.17	2.73	0.09
Monthly family income >Rs. 10,000	0.45	-	-	1.06	Rej	3.57	-	-	2.06	-	-
Healthcare decision-Collective decision	-0.09	-	-	-1.13	Rej	0.65	-	-	0.72	-	-
Healthcare decision-Self	0.05	-	-	-0.80	Rej	3.68	-	-	2.52	-	-
Healthcare decision-Spouse	-0.76	-0.73	-	-0.09	Rej	1.25	-	-	1.10	-	-
Help from government healthcare facilities	-2.03	-2.01	-0.12	0.40	Rej	3.90	-	-	0.54	-	-
Help from private healthcare facilities	-0.84	-0.92	-	0.66	Rej	3.22	-	-	2.88	-	-
Distance of healthcare facility >5 km	0.87	0.85	0.01	0.81	Rej	8.04	-	-	3.45	-	-
Antibiotics used for ear-nose-throat	-1.02	-	0.00	0.61	Rej	1.23	-	-	0.39	-	-
Antibiotics used for eyes	1.72	1.79	0.85	12.00	Cnf	12.86	14.67	17.42	3.55	1.94	1.53
Antibiotics used for gastro-intestinal system	0.64	1.13	0.20	3.35	Tntv	6.66	2.43	-	2.36	1.44	-
Antibiotics used for injury or accident	-2.46	-2.34	0.00	-0.62	Rej	2.49	-	-	0.00	-	-
Antibiotics used for musculoskeletal disorders	-1.40	-	0.00	0.88	Rej	1.28	-	-	0.18	-	-
Antibiotics used for respiratory system	-1.49	-1.00	-0.29	0.69	Rej	6.17	-	-	1.60	-	-
Antibiotics used for surgery	0.15	-	0.00	-0.95	Rej	1.09	-	-	0.73	-	-
Total no. of persons who consumed antibiotics->1 person	0.49	-	0.37	5.61	Cnf	3.84	2.77	0.00	4.16	1.06	0.00
Total no. of days antibiotics consumed-6 to 10 days	0.84	1.20	0.00	-0.14	Rej	2.35	-	-	0.96	-	-
Total no. of days antibiotics consumed >10 days	0.58	1.16	0.00	5.39	Cnf	5.04	0.04	3.61	1.94	0.00	0.70
Total no. of tablet/syrup of antibiotics consumed >2 doses	0.53	-	0.00	5.03	Cnf	6.75	0.00	3.24	2.21	1.90	1.23
Antibiotics medicines were affordable-Yes	0.01	-	-	0.54	Rej	0.00	-	-	1.95	-	-
Overall money spent on purchasing antibiotic medicines >Rs. 200	-0.07	-	-	1.35	Rej	1.25	-	-	3.20	-	-
Perceived effect of antibiotic medicines on health outcome-Not cured/deteriorated	-1.48	-1.54	-	1.15	Rej	1.11	-	-	0.15	-	-
Problems after consuming antibiotic medicines-Yes	-0.03	-	0.00	0.56	Rej	2.39	-	-	0.39	-	-
Completed dose of antibiotic medicine prescribed by doctor-Yes	-0.97	-0.88	-0.02	3.55	Tntv	6.71	5.21	-	2.58	1.64	-
Effects/consequences of incomplete dose of antibiotic medicines-Not aware	0.40	-	0.00	-0.49	Rej	3.84	-	-	2.89	-	-
Effects/consequences of incomplete dose of antibiotic medicines-Incomplete recovery	0.24	-	0.00	-0.21	Rej	3.84	-	-	1.40	-	-
Perception of buying medicines directly from medicine shop/pharmacy-Useful	8.19	7.99	4.85	77.92	Cnf	100.00	100.00	100.00	100.00	100.00	100.00
Antibiotics are beneficial for human beings-Beneficial	-2.02	-2.03	-0.59	-0.28	Rej	0.71	-	-	2.86	-	-

Nine nonrej variables were selected by Boruta: f1_Block2, f2_Others, eyes_Yes, persons_2gt1persons, days_2gt10days, dose_2gt2doses, f13_Useful, f11_Yes, gis_Yes; Seven cnf variables were selected by Boruta: f1_Block2, f2_Others, eyes_Yes, persons_2gt1persons, days_2gt10days, dose_2gt2doses, f13_Useful. OTC, over-the-counter; RF, random forest; XGBtree, extreme gradient boosting tree; Cnf, confirmed important; Rej, rejected; Tntv, tentatively important, nonrej, non-rejected (7 cnf+2 Tntv variables=9 variables); RS, Indian rupee.

**Table 3.** Evaluation of machine learning models using test data

Prediction models	AUROC (95% CI)	Log-loss	Accuracy	Sensitivity	Specificity	F1-score	Balanced accuracy
Full logistic regression	0.904 (0.843, 0.965)	0.386	0.879	0.919	0.863	0.809	0.891
Stepwise logistic regression	0.905 (0.845, 0.964)	0.378	0.864	0.892	0.853	0.786	0.872
Lasso regression	0.902 (0.833, 0.971)	0.326	0.886	0.971	0.857	0.815	0.914
RF (29 predictors)	0.919 (0.863, 0.975)	0.291	0.909	0.949	0.892	0.861	0.921
RF+Boruta (nonrej) (9 predictors)^1^	0.918 (0.859, 0.976)	0.283	0.886	0.971	0.857	0.815	0.914
RF+Boruta (cnf) (7 predictors)^2^	0.928 (0.878, 0.978)	0.360	0.909	0.927	0.901	0.864	0.914
XGB tree (29 predictors)	0.918 (0.859, 0.977)	0.298	0.909	0.949	0.892	0.860	0.921
XGB tree+Boruta (nonrej) (9 predictors)^1^	0.930 (0.883, 0.976)	0.303	0.894	0.884	0.899	0.844	0.891
XGB tree+Boruta (cnf) (7 predictors)^2^	0.934 (0.891, 0.978)	0.279	0.909	0.927	0.901	0.864	0.914

AUROC, area under the curve; CI, confidence interval; cnf, confirmed important (7 predictors); nonrej, non-rejected (7 cnf+2 Tntv variables) (9 predictors); RF, random forest; XGB, extreme gradient boosting.

1 9 nonrej predictors: f1_Block2, f2_Others, eyes_Yes, persons_2gt1persons, days_2gt10days, dose_2gt2doses, f13_Useful, f11_Yes, gis_Yes.

2 7 cnf predictors: f1_Block2, f2_Others, eyes_Yes, persons_2gt1persons, days_2gt10days, dose_2gt2doses, f13_Useful.

## Supplementary materials

Supplementary Material 1. List of predictor/independent variables
