# Supplementary material for: Predicting over-the-counter antibiotic use in rural Pune, India, using machine learning methods
Source: Epidemiol Health. 2024 Apr 13;46:e2024044. doi: 10.4178/epih.e2024044 (PMC11417445; doi:10.4178/epih.e2024044)
Supplement: Supplementary Material 1. — List of predictor/independent variables [file epih-46-e2024044-Supplementary-1.docx]

**Supplementary material**

**List of predictor/independent variables**

| **Variable name** | **Type of variable** | **Variable description** |
| --- | --- | --- |
| f1_Block2 | Binary | Rural |
| f2_Others | Binary | Social group - Others |
| f3_2gt10.000 | Binary | Monthly family income > Rs. 10.000 |
| f4_Collective.decision | Binary | Healthcare decision - Collective decision |
| f4_Self | Binary | Healthcare decision - Self |
| f4_Spouse | Binary | Healthcare decision - Spouse |
| f5_Govt | Binary | Help from Government healthcare facilities |
| f5_Pvt | Binary | Help from Private healthcare facilities |
| f6_2gt5.km | Binary | Distance of healthcare facility > 5 km |
| ent_Yes | Binary | Antibiotics used for ENT |
| eyes_Yes | Binary | Antibiotics used for Eyes |
| gis_Yes | Binary | Antibiotics used for Gastro-intestine system |
| injuryaccident_Yes | Binary | Antibiotics used for Injury or accident |
| musculoskeletal_Yes | Binary | Antibiotics used for Musculoskeletal |
| respiratory_Yes | Binary | Antibiotics used for Respiratory system (RTI/URTI) |
| surgery_Yes | Binary | Antibiotics used for Surgery |
| persons_2gt1persons | Binary | Total no. of persons consumed antibiotics >1 person |
| days_6to10days | Binary | Total no of days antibiotics consumed: 6 to 10 days |
| days_2gt10days | Binary | Total no of days antibiotics consumed > 10 days |
| dose_2gt2doses | Binary | Total no of tablets/syrups of antibiotics consumed > 2 doses |
| f7_Yes | Binary | Antibiotics medicines were affordable - Yes |
| f8_2gt200 | Binary | Overall money spent on purchasing antibiotic medicines > Rs. 200 |
| f9_Notcured_Deteriorated | Binary | Perceived effect of antibiotic medicines on health outcome - Not cured/ deteriorated |
| f10_Yes | Binary | Problems after consuming medicines - Yes |
| f11_Yes | Binary | Completed dose of antibiotic medicine prescribed by doctor - Yes |
| f12_Notaware | Binary | Effects/consequences for incomplete dose of antibiotic medicines - Not aware |
| f12_Notfullyrecovered | Binary | Effects/consequences for incomplete dose of antibiotic medicines - Not fully recovered |
| f13_Useful | Binary | Practice of buying medicines directly from medicine shop/pharmacy - Useful |
| f14_Useful | Binary | Antibiotics are useful for human beings - Useful |
